# Effects on Autophagy of Moxibustion at Governor Vessel Acupoints in APP/PS1double-Transgenic Alzheimer's Disease Mice through the lncRNA Six3os1/miR-511-3p/AKT3 Molecular Axis

**DOI:** 10.1155/2022/3881962

**Published:** 2022-10-05

**Authors:** Yu-Mei Jia, Cai-Feng Zhu, Ze-Yu She, Meng-Meng Wu, Yang-Yang Wu, Bing-Yuan Zhou, Na Zhang

**Affiliations:** ^1^Graduate School of Anhui University of Traditional Chinese Medicine, Hefei 230038, China; ^2^Second Affiliated Hospital of Anhui University of Traditional Chinese Medicine, Hefei 230061, China

## Abstract

**Objective:**

To explore the effect and mechanism of moxibustion at acupoints of the governor vessel on lncRNA Six3os1 in amyloid precursor protein/presenilin1 (APP/PS1) double-transgenic Alzheimer's disease (AD) mice.

**Methods:**

Twenty-four specific pathogen-free and APP/PS1 double-transgenic male mice were randomly allocated into the AD model and moxibustion groups, with 12 cases in each group. Twelve syngeneic C57BL/6J mice were selected as the control group. Mice in the moxibustion group received aconite cake-separated moxibustion at the Baihui acupoint. Suspension moxibustion was applied at Fengfu and Dazhui for 15 minutes each day. All treatments were conducted over two weeks. Control and AD model mice were routinely fed without any intervention. Behavioral observation tests were conducted before and after the intervention. The autophagosome in the hippocampus was observed using transmission electron microscopy. Immunohistochemistry was performed to detect A*β*1-42 expression. LC3B and P62 expressions were evaluated by immunofluorescence. The expression levels of the lncRNAs Six3os1, miR-511-3p, and AKT3 were detected by qRT-PCR. The differential expression of PI-3K, AKT3, mTOR, LC3B-II/I, and P62 proteins in the hippocampus was detected by western blot. The dual-luciferase assay was undertaken to examine the targeting relationships of the lncRNAs Six3os1, miR-511-3p, and AKT3.

**Results:**

Compared with the control group, the AD model showed higher escape latency in the Morris Water Maze and reduced autophagic vacuoles in the cytoplasm of hippocampal neurons (both *p* < 0.01). Compared with the control group, the AD model showed higher expression of A*β*1-42, the lncRNAs Six3os1, PI-3K, mTOR, P62, and AKT3 protein (all *p* < 0.01); but lower mir-511-3p and LC3B (both *p* < 0.01). Compared with the AD model group, the moxibustion group had a shorter escape latency, more autophagic bubbles in the hippocampus, and lower expression of positive A*β*1-42, the lncRNAs Six3os1, PI-3K, mTOR, P62, and AKT3 protein (all *p* < 0.01). In contrast, the levels of miR-511-3p and LC3B proteins were considerably increased in the moxibustion group compared to the AD model group (both *p* < 0.01). Based on the dual-luciferase assay, there was a targeting link among the lncRNAs Six3os1, miR-511-3p, and AKT3.

**Conclusion:**

Moxibustion at acupoints of the governor vessel can suppress the lncRNA Six3os1 expression, promote cell autophagy, accelerate A*β*1-42 clearance and alleviate cognitive dysfunction of AD mediated by the PI3K/AKT/mTOR signaling pathway through the lncRNA Six3os1/miR-511-3p/AKT3 axis.

## 1. Introduction

Alzheimer's disease (AD) is a multifactorial and irreversible neurodegenerative disease that accounts for 50–70% of dementia cases [1]. Pharmacological therapies used to treat AD can relieve symptoms but do not reverse disease progression [2]. The failure of a range of clinical agents has led some to question the amyloid-*β* (A*β*) pathophysiological hypothesis of AD [3]. However, anti-A*β* drugs such as aducanumab and ALZ-801 have shown encouraging outcomes in phase 3 trials and have confirmed amyloid as a viable therapeutic target [4, 5]. Studies have established that dysfunctional autophagy is involved in neurodegenerative disease and that its induction can accelerate the clearance of abnormally accumulated A*β*, thereby improving cognitive function in AD [6]. Furthermore, an increasing number of studies have revealed that autophagy is implicated in the etiology and progression of AD [7, 8]. Therefore, the identification of key regulators of autophagy is essential for AD treatment.

LncRNAs are noncoding RNAs > 200 nucleotides in length that can be widely distributed in the nucleus; they have emerged as critical regulators of numerous basic biological activities [9]. Studies have shown that lncRNAs can be represented as molecular sponges to target miRNAs and influence cell autophagy directly [10]. Xu et al. found that overexpression of the lncRNA H19 affects the normal activity of the PI3K/Akt/mTOR signaling pathway, impairing impair cell survival and increasing cell autophagy.

Our earlier study found that moxibustion effectively treated AD by improving patient cognitive function and daily tasks. Underlying these clinical effects, moxibustion may act by inhibiting PI3K/AKT/mTOR and P38 MAPK signaling pathways to enhance cell autophagy and accelerate A*β* clearance [11–13]. We examined gene expression in the hippocampal tissue of APP/PS1 double-transgenic mice by utilizing high-throughput sequencing technology. We aimed to determine the differential expression of lncRNAs and mRNAs before and after the moxibustion treatment. Functional enrichment analyses were also performed to enrich the PI3K/AKT/mTOR signaling pathway to construct a ceRNA regulation network and eventually screen out the crucial lncRNA Six3os1. Next, we evaluated the specific effects and mechanisms of moxibustion in modulating the PI3K/AKT/mTOR signaling pathway through lncRNA Six3os1 to provide reliable molecular markers and targets for the clinical diagnosis and treatment of AD.

## 2. Materials and Methods

### 2.1. Animal Models

Six-month-old APP/PS1 double transgenic and specific pathogen-free male AD mice were provided by Nanjing Junke Bioengineering Co., Ltd. [License: SCXK (Su) 2020-0009]. The average body weight was 28 ± 2 g. The animals were raised in a clean animal room at the Science Experimental Center of the Anhui University of Chinese Medicine. Each animal was kept in a separate cage clarified at 23 ± 2°C, 50 ± 5% humidity, and in a 12-hour light-dark cycle. Approval for the study was provided by the Anhui University of Chinese Medicine (NO: AHUCM-mouse-2021042).

The Morris water maze spatial learning test was performed one week following adaptive feeding; animals who did not swim or exhibited significant differences in test scores from other mice were excluded from the experiment. The remaining 24 animals were randomly but evenly divided into the AD model and moxibustion groups. Twelve healthy wild-type C57BL/6J mice were simultaneously screened for the control group. The experiments were conducted according to the requirements of the Caring for Laboratory Animals guidelines issued by the Ministry of Science and Technology in 2006.

### 2.2. Reagents and Instruments

PI3K (ab86714, Abcam), AKT3 (bs-5146R, Bioss), mTOR (2972s, CST), LC3B (bs-2912R, Bioss), P62 (18420-1-AP, Triple Eagle), western removal buffer of primary antibody and second antibody (P0025, Beyotime), RIPA cell lysate (P0013B, Beyotime), ECL hypersensitive luminescence kit (34095, Thermo), goat anti-mouse IgG secondary antibody (ZB-2305, Zsbio), goat anti-rabbit IgG secondary antibody (ZB-2301, Zsbio), goat anti-rabbit IgG (FITC) (B029, Ebiogo), sheep serum block (B010, Ebiogo), anti-fluorescence quench blocking agent (containing DAPI) (B024, Ebiogo), TRIzol (15596026, Life Technologies), and hematoxylin (BA-4041, BaSO).

EPS 300 electrophoresis instrument (Tanon), VE-180 electrophoresis tank (Tanon), JW-3021HR high-speed refrigerated centrifuge with 6.8cm centrifugal radius (Anhui Jiawen Instrument Equipment), JEM1400 flash transmission electron microscope (Jieou Lu, Beijing), PIKOREAL 96 fluorescence quantitative PCR instrument (Thermo), OD1000+ ultra-micro spectrophotometer (Nanjing Wuyi), CX41 microscope (Olympus), RM2016 Leica microtome (Leica), Pannoramic MIDI digital section scanner (3DHISTECH), and a 1319A digital thermometric indicator(Shanghai TES).

### 2.3. Experimental Intervention

The acupoints Baihui, Dazhui, and Fengfu were selected according to the Nomenclature and Location of Acupuncture Points for Laboratory Animals [14]. Animals were shaved at the intervention site and acupoints were marked. Several aconite cakes with a diameter of ∼1 cm and a thickness of 4–6 mm were prepared in advance and multiple small holes were made on their surface with a toothpick. Mice were secured in a restraint with the head and neck exposed. In the moxibustion-treated group, the moxibustion bar was lit and the lit end was placed on the aconite cake, which was then positioned at Baihui for 15 minutes every day. Dazhui and Fengfu were treated with suspension moxibustion for 15 minutes per day at a distance of 2–3 cm from the skin. To control for radiant heat, one end of a temperature probe was affixed next to the Fengfu acupoint and the moxibustion temperature was maintained at 44–46°C (the same protocol was followed at Dazhui acupoints). The control and AD model groups were routinely housed under comparable conditions but received no intervention. All mouse groups were treated once daily for two weeks. Trained professionals from the Anhui Acupuncture Hospital performed the procedures.

### 2.4. Outcome Variables

#### 2.4.1. Morris Water Maze Test of Spatial Localization

The water maze test was performed before and after the intervention. The laboratory was sheltered from light, and the room temperature was maintained at 24–25°C with the water temperature at 24 ± 2°C. Mice were continuously trained for four days before data collection. On the first day, the mice were placed in the water for two minutes to adapt to the surroundings. Each mouse received two training sessions, separated by 4 hours, per day from the second day. Each time, they were introduced to the water from a different point. The latency time (from placement until arrival at the platform) was recorded. If the mouse did not find the platform within 2 minutes, the latency was recorded as 2 minutes. The mice were permitted to stay on the platform for 30 seconds whether or not they found the platform within 2 min. Following the training, the mice were tested to establish a baseline and again after the treatment.

#### 2.4.2. Hippocampal Autophagy

Brains were harvested from six mice and the hippocampi were isolated; harvesting was performed on an ice plate. The hippocampal CA1 region was excised and stored in an electron microscopy solution for subsequent electron microscopy analysis; the remaining hippocampal tissues were preserved at −80°C for gene and protein quantification. Several isolated fresh sections (∼1 mm^3^) of the hippocampal CA1 region were rinsed, fixed, dehydrated, and embedded for ultrathin sectioning (60 nm thickness). Three sections were taken from the mice of each group. The morphology of neuronal cell structures, autophagic vacuoles, autolysosomes, and lysosomes was examined and photographed using the JEM1400 transmission electron microscope after double staining (lead and uranium staining). Three to five fields were randomly selected from each section for analysis.

#### 2.4.3. A*β*1-42 Protein Expression

Three mice from each group were randomly selected and anesthetized by intraperitoneal injection with 0.3% pentobarbital sodium (30 mg/kg). The brains were fixed after perfusion with 4% paraformaldehyde. Sections of 4 *μ*m thickness were made from prepared paraffin blocks, then deparaffinized and hydrated for antigen retrieval. The sections were then rinsed ×3 in phosphate-buffered saline ×5 min, blocked, and incubated in A*β* (1 : 1000) primary antibody overnight at 4°C. The secondary antibody (1 : 5000) was added dropwise to the sections for incubation at 37°C for 40 minutes before rinsing. DAB was added dropwise to the sections and the chromogenic time was adjusted under the microscope. The sections were washed following appropriate color development. The sections were rewashed after hematoxylin counterstaining for two to five minutes, after which the sections were washed after blueing with lithium carbonate solution for 30 seconds. After dehydration and xylene-induced tissue transparency, neutral gum was then added and the sections were coverslipped for imaging. The images were observed and captured under a high-power (×400) microscope to determine the mean absorbance values of the positive staining.

#### 2.4.4. Hippocampal Expression of lncRNAs Six3os1, miR-511-3p, and AKT3 in APP/PS1 Mice

–80°C frozen hippocampal tissues weighing 50–60 mg were weighed, chopped, and total RNA was extracted using TRIzol-chloroform-isopropanol-ethanol. According to the instructions, RNA was reverse transcribed into cDNA using a PrimeScript™ RT reagent Kit with gDNA Eraser (TaKaRa). The PCR reaction system consisted of 5 *μ*L of 2 × SYBR Green mixture, 1 *μ*L of each upstream and downstream primers, 1 *μ*L cDNA, and 2 *μ*L of nuclease (10 *μ*L of the final mixture). The PCR parameters for lncRNAs Six3os1, miR-511-3p, and AKT3 were as follows: 95°C for 1 minute, 95°C for 20 seconds, and 60°C for 1 minute. The fluorescence signals were acquired over 40 cycles with three technical replicates per sample. *β*-actin was used as the internal reference gene and the results were analyzed by using the 2^−ΔΔCt^ method after appropriate quality checking (e.g. melt curves). Primer sequences are shown in Table 1.

#### 2.4.5. LC3B and P62 Hippocampal Expression

Three mice in each group were randomly selected and their brains were harvested and cut into two along the sagittal axis. The fresh brain tissues were placed on a frozen plate and embedded in OCT until the tissues were covered entirely, following which the tissues were stored at −80°C refrigerators until frozen. The brains were sectioned and rinsed. Antigen retrieval was done in a pressure-cooker then goat serum blocking solution was added dropwise and incubated at 37°C. The primary antibody (LC3B/P62) was added dropwise and incubated at 37°C for 60 minutes. The secondary antibody (goat anti-rabbit; 1 : 400) was added dropwise, capped, and incubated at 37°C in the dark for 30 minutes. Then, an anti-fluorescent quench blocking agent was added (containing DAPI) and the fluorescent sections were scanned by using Pannoramic MIDI.

#### 2.4.6. Hippocampal Expression of PI-3K, AKT3, mTOR, LC3B-II/I, and P62 Protein

Approximately 100 mg of hippocampal tissue, frozen at −80°C, was lysed in 1 ml of RIPA cell lysis buffer + 1 mM PMSF protease inhibitor. The samples were centrifuged at 1,200 RCF for 10 min at 4°C, and the supernatants were collected for protein extraction. The proteins were tested in seven consecutive steps including denaturation, loading electrophoresis, transmembrane, blocking with 5 percent fat-free milk powder for 2 hours at room temperature, and incubation with primary antibodies (PI-3K, mTOR, and LC3B-II/I at 1 : 1000; AKT3 at 1 : 500) overnight at 4°C. The next day the membranes were washed and incubated with secondary antibodies (goat anti-mouse -IgG and goat anti-rabbit IgG at 1 : 1000) at room temperature for 1.2 hours. The ECL luminescence kit was used for detection on film with appropriate exposure and film development. Image *J* software was used to analyze film strips. GAPDH was used as the reference protein.

#### 2.4.7. The Targeting Relationship of lncRNAs Six3os1, miR-511-3p, and AKT3

We used the starbase database (https://starbase.sysu.edu.cn/) to predict binding sites and construct dual-luciferase reporter vectors, including Six3os1-wt, Six3os1-mut, AKT3-wt, and AKT3-mut. 293T cells and target plasmids previously prepared for transfection were dispensed into 96-well plates at 50–70% confluence. The target plasmid was fully mixed with 5 pmol of miR-511-3p (Negative Control, NC) at room temperature (solution A), following which 10 *μ*L DMEM was mixed thoroughly with 0.3 *μ*L of transfection reagent (HANBIO product with a concentration of 0.8 mg/mL) at room temperature for 5 min (solution *B*). Solution *A* was thoroughly mixed with solution *B* at room temperature for 20 min. The cell media were refreshed before transfection, following which the transfection mixture was added to the mix and incubated at 37°C with 5% CO_2_. After six hours, the media were exchanged and the cells were incubated for 48 hours. Luciferase activity was assayed by following the instructions of the Promega Dual-Luciferase assay kit.

### 2.5. Statistical Analysis

SPSS version 23.0 was used for all statistical analyses. Data were expressed as mean ± standard deviation (mean ± SD). One-way analysis of variance was used to test differences between multiple groups and the least significant difference was used to examine group differences. Data that were non-normally distributed or showed signs of heteroscedasticity were analyzed by using the Kruskal-Wallis H test. Statistical significance was determined by *p* < 0.05.

## 3. Results and Discussion

### 3.1. Results

#### 3.1.1. Morris Water Maze

**Figure 1:**
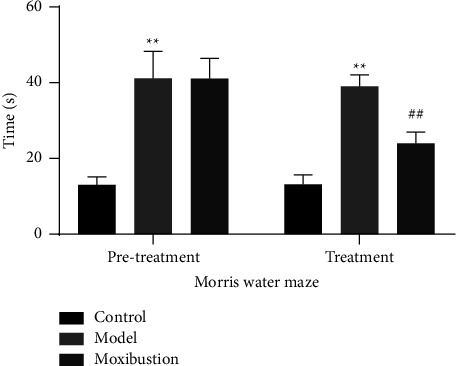
Comparison of Morris Water Maze escapes latency (mean ± SD, *n* = 12). Note: ^*∗∗*^indicates significant difference versus the control group, at *p* < 0.01; ^##^indicates significant difference versus the AD model group, at *p* < 0.01.

As shown in Figure 1, before treatment, the escape latency was markedly longer in the AD model group of the double transgenic mice compared with the control mice (*p* < 0.01). There was no significant difference between the AD model and moxibustion groups (*p* > 0.05). After the intervention, the escape latency of AD mice in the model group was longer compared with the control group (*p* < 0.01). The escape latency was substantially shorter in the moxibustion group than in the AD model group (*p* < 0.01).

#### 3.1.2. Hippocampal Autophagy

**Figure 2:**
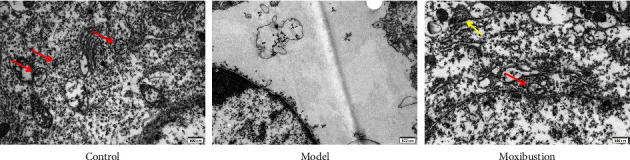
Cytoplasmic ultrastructural structures in the hippocampus. Note: the red arrow indicates autophagic vacuoles; the yellow arrow indicates autophagosome.

tAs shown in Figure 2, organelles were well arranged with clear and complete structures. More autophagic vacuoles and autophagosomes were observed in the hippocampal cytoplasm of the control mice. Deformed and atrophied organelles were detected in the AD model group and the autophagic vacuoles were significantly reduced. Organelles were abundant and autophagic vacuoles and autophagosomes were increased in the moxibustion group compared with the AD model group.

#### 3.1.3. A*β*1-42 Protein Expression

**Figure 3:**
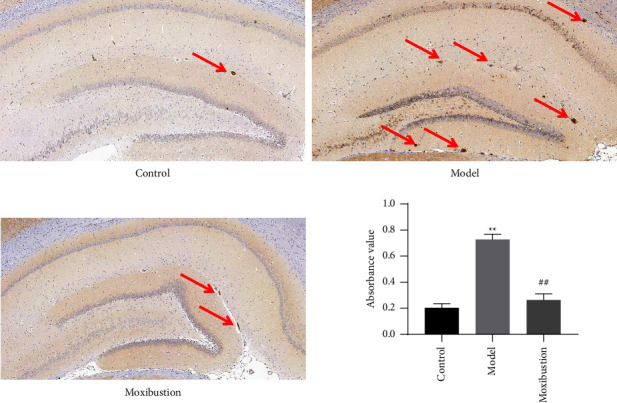
Hippocampal A*β*_1–42_ protein content (mean ± SD, *n* = 3). Note: arrows indicate senile plaques. ^*∗∗*^indicates significant difference versus the control group, at *p* < 0.01; ^##^indicates significant difference versus the AD model group, at *p* < 0.01.

As shown in Figure 3, A*β*_1–42_ protein content was markedly increased in the AD model group compared with the control group, as determined by immunohistochemistry (*p* < 0.01). A*β*_1–42_ content was significantly decreased in the moxibustion group compared to the AD model group (*p* < 0.01).

#### 3.1.4. Hippocampal Expression of lncRNAs Six3os1, miR-511-3p, and AKT3

**Figure 4:**
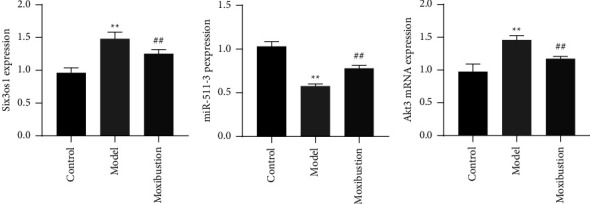
Expression of the lncRNAs Six3os1, miR-511-3p, and AKT3 in the hippocampus (mean ± SD, *n* = 6). Note: ^*∗∗*^indicates significant difference versus the control group, at *p* < 0.01; ^##^indicates significant difference versus the AD model group, at *p* < 0.01.

As shown in Figure 4, compared with the control group, in the AD model the expression of the lncRNAs Six3os1 and AKT3 was increased while miR-511-3p expression was lower, as determined by qRT-PCR (both *p* < 0.01). In contrast, compared to the AD model group, in the moxibustion group, the expression of the lncRNAs Six3os1 and AKT3 was decreased and miR-511-3p expression was significantly increased (both *p* < 0.01).

### 3.2. Hippocampal Expression of LC3B and P62

**Figure 5:**
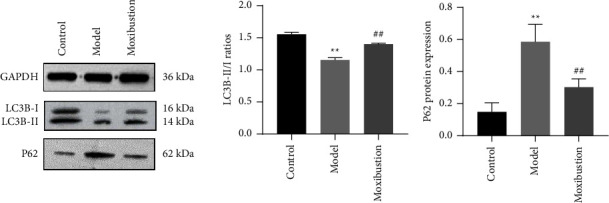
LC3B-II/I ratio and P62 hippocampal expression (mean ± SD, *n* = 6). Note: ^*∗∗*^indicates significant difference versus the control group, at *p* < 0.01; ^##^indicates significant difference versus the AD model group, at *p* < 0.01.

As shown in Figure 5, compared with the control group, in the AD model the LC3B-II/I ratio was markedly decreased, while P62 was significantly increased, as determined via WB (both *p* < 0.05). Compared with the model group, in the moxibustion group, the LC3B-II/I ratio was greatly increased, while P62 expression was increased (both *p* < 0.05).

**Figure 6:**
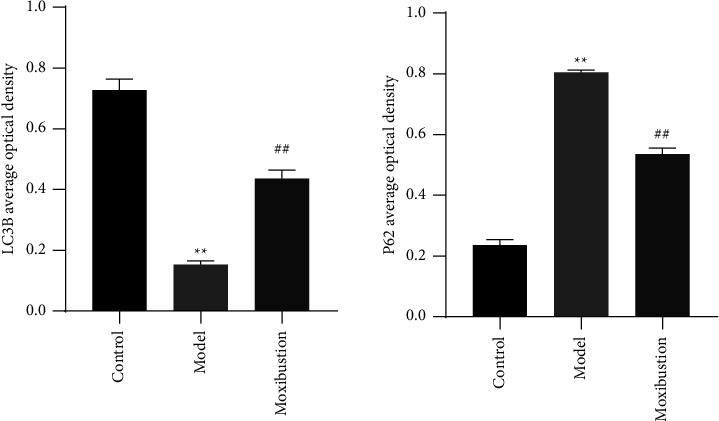
Hippocampal expression of LC3B and P62 (mean ± SD, *n* = 3). Note: Nuclei are in blue and LC3B/P62 are in green. (a) LC3B average optical density; (b) P62 average optical density. ^*∗∗*^indicates significant difference versus the control group, at *p* < 0.01; ^##^indicates significant difference versus the AD model group, at *p* < 0.01.

As shown in Figure 6, the fluorescent LC3B signal was attenuated in the AD model and moxibustion groups compared with the control group. At the same time, P62 was significantly enhanced in the AD model group (both *p* < 0.01). Compared with the model group, in the moxibustion group, LC3B fluorescence was significantly enhanced and P62 was greatly attenuated (both *p* < 0.01).

### 3.3. Hippocampal Protein Expression of PI3K/AKT/mTOR Pathway-Related PI3K, AKT3, and mTOR

**Figure 7:**
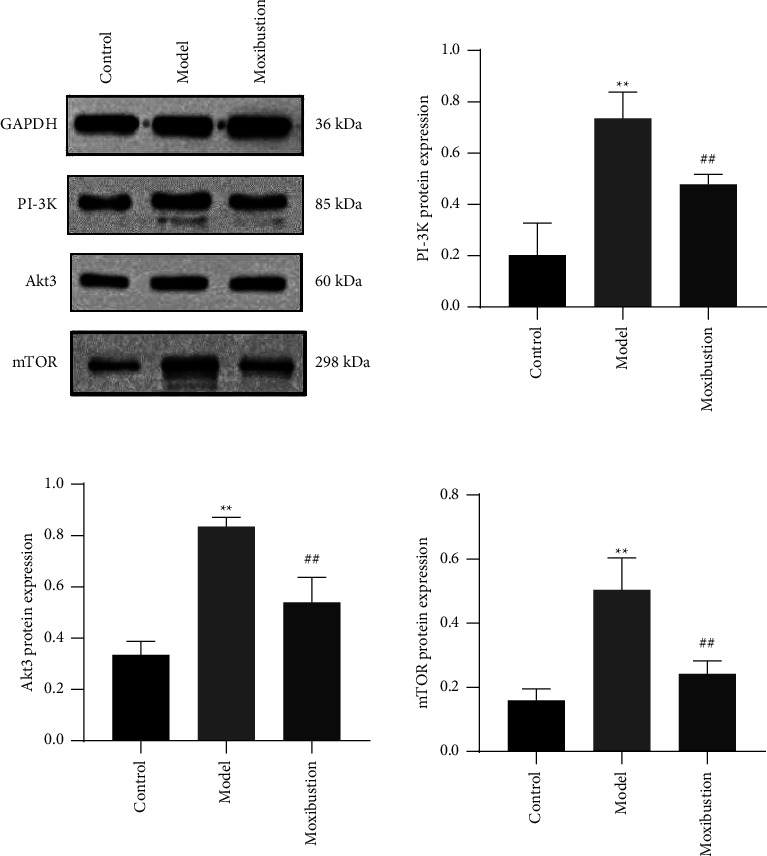
Hippocampal expression of PI3K, AKT3, and mTOR proteins (mean ± SD, *n* = 6). Note: ^*∗∗*^indicates significant difference versus the control group, at *p* < 0.01; ^##^indicates significant difference versus the AD model group, at *p* < 0.01.

As shown in Figure 7, the expressions of PI3K, AKT3, and mTOR proteins were significantly increased in the model group compared to the control group, as demonstrated by WB (all *p* < 0.01). Conversely, compared with the model group, the moxibustion group showed lower expression of PI3K, AKT3, and mTOR proteins (all *p* < 0.01).

## 4. Binding Site Prediction and Results of the Dual-Luciferase Assay

**Figure 8:**
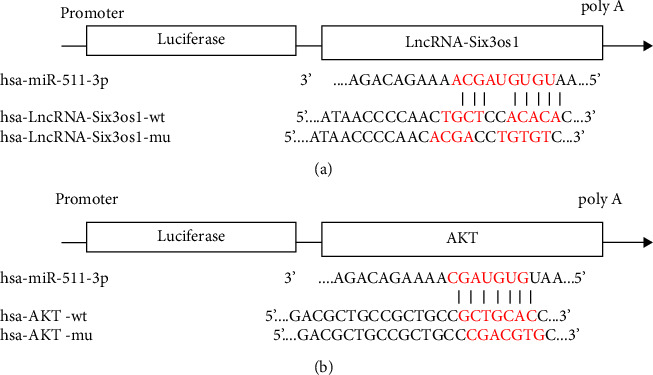
Schematic diagram of the predicted binding site: (a) Schematic diagram of hsa-miR-511-3p binding to lncRNA-Six3os1 target sites; (b) schematic diagram of hsa-miR-511-3p binding to AKT target sites.

As shown in Figure 8, starbase binding sites predicted complementary binding sites for the miR-511-3p and the 3′UTR of lncRNA-Six3os and AKT and constructed the mutation sites of lncRNA-Six3os-3′UTR and AKT-3′UTR.

**Figure 9:**
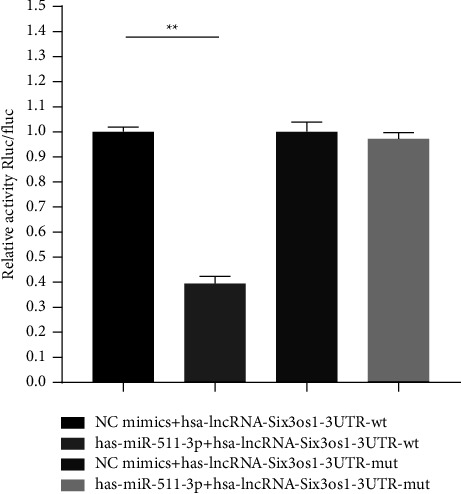
Dual-luciferase activity analysis. Group 1. NC mimics + hsa-lncRNA-Six3os1-3UTR-wt; Group 2. has-miR-511-3p + hsa-lncRNA-Six3os1-3UTR-wt; Group 3. NC mimics + has-lncRNA-Six3os1-3UTR-mut; Group 4. has-miR-511-3p + hsa-lncRNA-Six3os1-3UTR-mut (^∗∗^indicates a statistically significant difference at *p* < 0.01).

As shown in Figure 9, compared with the NC group, the miR-511-3p significantly downregulated the luciferase expression of lncRNA-Six3os-3′UTR-wt (*p* < 0.01), while miR-511-3p failed to downregulate the luciferase expression of lncRNA-Six3os-3'UTR-mut after the mutation (*p* > 0.05).

**Figure 10:**
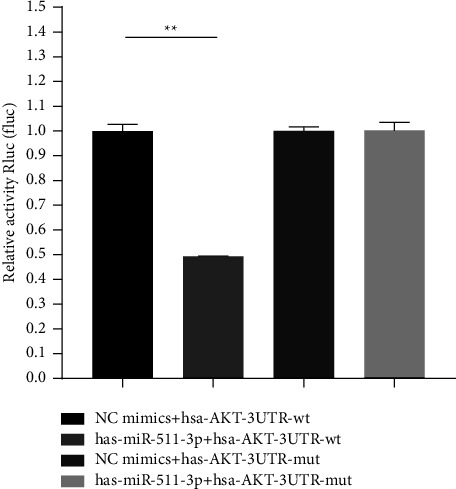
Dual-luciferase activity analysis. Group 1. NC mimics + hsa-AKT-3UTR-wt; Group 2. has-miR-511-3p + hsa-AKT-3UTR-wt; Group 3. NC mimics + has-AKT-3UTR-mut; Group 4. has-miR-511-3p + hsa-AKT-3UTR-mut. (^∗∗^indicates a statistically significant difference at *p* < 0.01).

As shown in Figure 10, compared with the NC group, the miR-511-3p significantly downregulated the luciferase expression of AKT-3'UTR-wt (*p* < 0.01), while miR-511-3p failed to downregulate the luciferase expression of AKT-3′UTR-mut after the mutation (*p* > 0.05).

## 5. Discussion

AD is a type of dementia. In Chinese Medicine, several factors are recognized in older individuals as being pathogenic for AD including dysfunction of zang-fu organs, marrow sea deficiency, promotion of kidney Yang, and poor function of warmth. Professor CAI Shengchao, a renowned traditional Chinese physician posited the hypothesis underlying our study; that is, that the governor vessel connects to brain function, inline with the heart and kidney, and passes through the Ren Channel, where all are organically connected with the function of the house of the soul. Therefore, it was proposed that dementia treatment should start from the shen-brain-governor vessel-kidney-Ren Channel axis [15]. In our experiment, we selected the three acupoints Baihui, Fengfu, and Dahui from the governor vessel, with the intent of thriving the governor vessel, filling the marrow sea, resolving the phlegm, and opening the brain orifices.

The pathogenesis of AD is complicated and includes the abnormal accumulation of A*β*, Tau protein hyperphosphorylation, and neuroinflammation [16, 17]. Furthermore, the deposition of amyloid-*β* produces further pathogenic cascades that underlie the primary mechanisms for AD etiology and progress [18]. Gene mutations in amyloid precursor protein (APP), presenilin-1 (PS-1), and presenilin-2 (PS-2) can mediate the A*β* hypersecretion and eventually deposit to form senile plaques, which trigger neuronal damage and death [19]. In our study, we observed that in the AD model, the escape latency in the Morris Water Maze was significantly prolonged and A*β*_1–42_ expression was significantly higher than in the control group. The moxibustion intervention reduced A*β*_1–42_ deposition in AD mice and improved spatial memory.

Autophagy is a primary metabolic process in eukaryotic cells that utilize lysosomes to degrade damaged organelles and aberrant proteins to maintain cell homeostasis [20]. As a landmark protein of autophagy, LC3B is involved in forming early autophagic vacuoles and can reflect the extent of autophagy [21]. The autophagic substrate P62 is continuously consumed during autophagy formation and reflects autophagic activity [22]. It has been demonstrated that activation of autophagy can reduce A*β* levels [23]. For example, Chen et al. reported several biochemical alterations that can enhance autophagic activity, including downregulation of miR-331-3p and miR-9-5p which markedly attenuated the accumulation of A*β* in mice with early AD [24]. Wu et al. reported that SIRT5 overexpression can ameliorate AD progression *in vitro* and *in vivo* by activating autophagic mechanisms to clear A*β* protein [25].

AKT, as a serine/threonine protein kinase, is a central effector molecule of the phosphoinositide 3-kinases/protein kinase B (PI3K/AKT) signaling pathway with its location at its hub [26]. AKT can influence downstream mTOR signaling, thereby contributing to the PI3K/AKT/mTOR pathway. The PI3K/AKT/mTOR signaling pathway is one of the critical pathways that regulate autophagy; inhibition of this pathway can activate autophagy in the AD mouse model [27]. Here we found that AD mice exhibited reduced autophagic vesicles and autophagosomes, indicating defective autophagy and promotion of A*β* deposition. The expression of PI3K, AKT3, mTOR, and P62 protein was decreased after moxibustion compared with the AD model group. In contrast, the autophagy marker protein LC3B was increased, indicating that moxibustion might inhibit the PI3K/KT/mTOR signaling pathway to promote autophagy and reduce A*β* deposition; this, in turn, improved the cognitive function of the treated mice. This outcome was consistent with prior research [11].

Several studies have demonstrated that lncRNA is involved in essential physiological processes such as hippocampal development, neuronal differentiation, and brain aging in mice [28]. The previous sequencing had screened out Six3os1, a critical differential gene lncRNA with high expression in the AD mouse hippocampus. It is known that Six3os1 can operate as a molecular scaffold to recruit histone-modifying enzymes to homeodomain factor Six3 target genes, which can regulate the activity of the related protein-coding genes and play a vital role in controlling neurodevelopment [29]. Geniposide might upregulate Six3os1 and attenuate the depressive-like-induced oxidative stress in mice through the miR-511-3p/Fezf1/AKT axis [30]. Combined with the ceRNA hypothesis, miR-511-3p was inferred as the target gene of lncRNAs Six3os1 and AKT. The software predicted its binding site and we validated it using a dual-luciferase reporter system. We showed that miR-511-3p strongly suppresses the expression of luciferin of wild-type lncRNAs Six3os1 and AKT, confirming that miR-511-3p is a target gene of the lncRNAs Six3os1 and AKT. As expected, these results were further corroborated by qRT-PCR with high expression of lncRNAs Six3os1 and AKT and low expression of miR-511-3p in the AD model group. Moxibustion lowered the expression of the lncRNAs Six3os1 and AKT and elevated miR-511-3p.

## 6. Conclusions

In summary, we speculated that moxibustion can regulate the lncRNA Six3os1 and inhibit the PI3K/Akt/mTOR signaling pathway via miR-511-3, thus modulating downstream target proteins to promote cell autophagy. Those effects contributed to the acceleration of A*β*_1–42_ clearance in the hippocampus, reduced neuronal damage, and alleviated cognitive dysfunction. These data provide support for the lncRNA Six3os1 as a molecular marker and target for the diagnosis and treatment of AD [31].

## Figures and Tables

**Table 1:** Primer sequences.

Gene	Primer sequence (5′⟶3′)	Amplicon size (bp)
*β*-actin	F: AGTGTGACGTTGACATCCGT R: TGCTAGGAGCCAGAGCAGTA	120

Six3os1	F: TCTTGAGTACCCCTAGCACT R: ATACGAAGATGGGCTTCCAG	88

miR-511-3p	F: ACACTCCAGCTGGGAATGTGTAGCAAAAGA R: TGGTGTCGTGGAGTCG	66

Akt3	F: CCCCTCAACAACTTCTCAGT R: CGTCCACTCTTCTCTTTCCT	150

## Data Availability

The data that support the findings of this study are available from the corresponding author upon reasonable request.
